# Editorial: Gene Regulation Explored by Systems Biology in Livestock Science

**DOI:** 10.3389/fgene.2022.859061

**Published:** 2022-04-08

**Authors:** Priyanka Banerjee, Aline Silva Mello Cesar, Andressa Oliveira De Lima

**Affiliations:** ^1^ Department of Animal Sciences, College of Agriculture, Auburn University, Auburn, AL, United States; ^2^ Department of Agri-Food Industry, Food and Nutrition, University of São Paulo, Piracicaba, Brazil; ^3^ Department of Genome Sciences, University of Washington, Seattle, WA, United States

**Keywords:** lncRNA, miRNA, transcriptomics, proteomics, methylation

In the livestock production system, comprehending animal health and production is increasingly necessary to meet the global food demands. The production of animals with desired traits is, therefore, a prerequisite. To meet future food requirements, the strategy targets vertical growth (increasing productivity) rather than expanding the livestock population. Improving productivity requires a better understanding of genes and genomes, and consequently, their influence on the trait of interest (i.e., production, reproduction, health and disease, or any other relevant traits in livestock), so that appropriate selection and breeding decisions can be implemented to improve livestock performance.

Over the past decade, much biological research has focused on gene expression and regulation by non-coding RNAs (ncRNAs), variants, cis- or distal regulatory elements (transcription factors, enhancers and silencers), histone modifications, and DNA methylation. Advancement in high-throughput technologies and cost reduction has enabled multi-omics studies to be performed on a large scale. Therefore, the crosstalk between multiple molecular layers is assessed by a systems biology approach that provides a systematic view of the regulatory mechanisms underlying complex traits. This approach proves beneficial for production systems (e.g., optimize animal nutrition, meat quality, or animal management) by selecting the desirable animals and integrating accurate breeding programs or innovative management systems.

For this research topic, we sought high-quality research papers describing novel insights into genetic and environmental factors that impact the mechanism and expression of production, reproduction and disease traits in livestock research. Research topics included the novel studies on the expression of genes, micro RNAs (miRNAs), long non-coding RNAs (lncRNAs), circular RNAs (circRNAs), proteins and methylome for the trait of interest in livestock. The final research topic has 12 articles covering the aforementioned aspects in bovine, ovine, caprine, porcine and poultry populations.

The quest for ideal therapeutic targets and biomarkers in disease traits in bovines has been riddled with many obstacles stemming from the molecular complexity of the disease and co-morbidities. Advances in omics technologies and the resulting amount of available data encompassing transcriptomics have created an opportunity to integrate omics datasets to identify molecular changes and represent data through networks in disease traits such as bovine respiratory disease (BRD) and mastitis. Hasankhani et al., identified key modules and potential hub genes from transcriptome data using co-expression networks that underlie BRD. With two different approaches of module–trait relationships and module preservation analysis, the authors identified eight candidate modules and 307 hub-genes involved in the immune response and BRD pathogenesis (Hasankhani et al.). Using a similar approach, Ghahramani et al., identified candidate genes and modules associated with mastitis in dairy cattle. Furthermore, they identified 360 meta genes within two modules by integrating microarray and RNA-Seq data. Additionally, the authors used attribute weighting and machine-learning methods to optimize predictive models using hub genes that were informative in *Escherichia coli* mastitis (Ghahramani et al.). These studies utilized a system’s level approach to provide a complete understanding of complex biological systems of BRD and mastitis beyond the molecular level in cattle.

Even though omics technologies reveal global changes in RNA modifications under various conditions, researchers also focused on diverse functions of specific RNAs, particularly ncRNAs and circRNAs, due to their potential in regulating gene expression. The ncRNAs are classified into several sub-classes, such as small non-coding RNAs, including miRNAs, small interfering RNAs, and lncRNAs, to name a few. Although lncRNAs biochemically resemble messenger RNAs (mRNAs), they do not template protein synthesis. Advances in computational biology and the evolution of sensitive RNA sequencing have facilitated the discovery of numerous lncRNAs and encouraged the study of their roles in livestock. An interesting study reported within this research topic involved the regulatory role of lncRNA in the transcriptome of abomasal lymph node tissue samples from adult Spanish Churra sheep after experimental infection with the gastrointestinal nematode *Teladorsagia circumcincta* (Chitneedi et al.). The authors identified ten differentially expressed lncRNAs between samples from animals that differ in infection resistance associated with signaling pathways like cellular growth, proliferation and development, cellular stress and injury, intracellular and second messenger signaling and apoptosis (Chitneedi et al.). In a separate study on sheep hair follicle development and morphogenesis, Xu et al., identified the *STAT3* gene partially inhibiting cell proliferation via direct negative regulation of *FST* gene expression.

miRNAs are small and highly conserved non-coding RNA molecules that orchestrate various biological processes through post-transcriptional expression regulation. A study by Kusama et al., aimed to characterize proteins and exosomal miRNAs in the uterine flushing of pregnant and non-pregnant cows after artificial insemination. The authors identified 336 proteins, of which 260 were more than two-fold higher in pregnant cows. The authors identified *SUGT1* as the best predictor for the presence of embryos in the uterus that altered protein composition and exosomal miRNA contents in the uterine fluid (Kusama et al.). In an independent miRNA study in cattle, bta-miR-150 was found to have a negative regulatory effect on the differentiation of bovine adipocytes and promoted proliferation, inhibited adipocyte differentiation, and reduced lipid droplet formation (Chen et al.). The research clarified the relationship between bta-miR-150 and adipocyte differentiation in cattle. Differentiation of intramuscular preadipocytes was also inhibited by overexpression of *KLF4* by targeting C/EBPβ in goats (Xu et al.). Since the adipose tissue in meat impacts the economic value of animals, these studies focusing on the molecular mechanisms underlying adipose tissue generation were primarily crucial for the beef production industry. As beef producers continue to improve efficiency, another crucial aspect of skeletal muscle mass impacts consumer acceptance of meat products. Sheng et al., used the proteomics approach to unravel the mechanism of myostatin in regulating cattle skeletal development.

Another study involved co-expression analysis focusing on miRNA-mRNA interaction networks (Yang et al.). An integrative analysis of the miRNA-mRNA expression profiles in the lungs of high- and low-altitude pigs (Tibetan pigs and Landrace pigs, respectively) identified molecular pathways and networks involved in the genetic adaptation of Tibetan pigs to hypoxic conditions (Yang et al.).

A separate group of lncRNA—circRNA—influences cellular physiology through various molecular mechanisms by modulating gene expression. A study by Li et al. identified circTAF8 regulating myoblast development and associated carcass traits in chicken.

One of the advantages of livestock research is that it proves to be an excellent physiological model for studies related to human health or disease. Studies reported by Chen et al., revealed the similarities and diversity of sperm methylation patterns among three commercial pig breeds and between humans and pigs. These findings elucidate the mechanism of male fertility and the changes in commercial traits that undergo strong selection. In a separate study by Xu et al., methylation changes in *SLC4A11* and *MFSD3* genes were analyzed after Deoxynivalenol toxicity in porcine cells that may contribute to the detection of biomarkers and drug targets.

Considering all the above studies, we believe that a holistic approach combining statistics, bioinformatics, and mathematical modeling to integrate and analyze large amounts of data generated and targeting some of the key regulators has shed light on mechanisms regulating the economically important traits in livestock. We hope that the reader will find this research topic a helpful reference for the state-of-the-art in the emerging field of livestock research.

